# Inferring single cell expression profiles from overlapped pooling sequencing data with compressed sensing strategy

**DOI:** 10.1093/nar/gkab581

**Published:** 2021-07-09

**Authors:** Mengting Huang, Yixuan Yang, Xingzhao Wen, Weiqiang Xu, Na Lu, Xiao Sun, Jing Tu, Zuhong Lu

**Affiliations:** State Key Laboratory of Bioelectronics, School of Biological Science and Medical Engineering, Southeast University, Nanjing 210096, China; State Key Laboratory of Bioelectronics, School of Biological Science and Medical Engineering, Southeast University, Nanjing 210096, China; State Key Laboratory of Bioelectronics, School of Biological Science and Medical Engineering, Southeast University, Nanjing 210096, China; Department of Statistics and Quantitative Economics, Institute of Economics, Shanghai Academy of Social Sciences, Shanghai 200020, China; State Key Laboratory of Bioelectronics, School of Biological Science and Medical Engineering, Southeast University, Nanjing 210096, China; State Key Laboratory of Bioelectronics, School of Biological Science and Medical Engineering, Southeast University, Nanjing 210096, China; State Key Laboratory of Bioelectronics, School of Biological Science and Medical Engineering, Southeast University, Nanjing 210096, China; State Key Laboratory of Bioelectronics, School of Biological Science and Medical Engineering, Southeast University, Nanjing 210096, China

## Abstract

Though single cell RNA sequencing (scRNA-seq) technologies have been well developed, the acquisition of large-scale single cell expression data may still lead to high costs. Single cell expression profile has its inherent sparse properties, which makes it compressible, thus providing opportunities for solutions. Here, by computational simulation as well as experiment of 54 single cells, we propose that expression profiles can be compressed from the dimension of samples by overlapped assigning each cell into plenty of pools. And we prove that expression profiles can be inferred from these pool expression data with overlapped pooling design and compressed sensing strategy. We also show that by combining this approach with plate-based scRNA-seq measurement, it can maintain its superiorities in gene detection sensitivity and individual identity and recover the expression profile with high precision, while saving about half of the library cost. This method can inspire novel conceptions on the measurement, storage or computation improvements for other compressible signals in many biological areas.

## INTRODUCTION

Single cells, as the basic components of life, are a new window to understand individual differences among cell (1,2). With the development of advanced technologies to capture single cells quickly and accurately (3,4), scientists can narrow down their view from bulk sequencing of thousands of cells, which averages out cellular difference, to subtle changes between individual cells (5). The elaborate atlas of single cells has shed light on multiple biological questions like revealing new cell types in cancers (6,7), investigating the dynamics of developmental processes (8), linkage and developmental trajectory of immune cells in cancer (9) and generating spatial transcriptomics landscape (10,11).

The two most popular methods for single-cell RNA-seq are plate-based methods and droplet-based methods. Plate-based methods like Smart-Seq2 (12,13) capture full-length transcripts by constructing sequencing library independently for each cell, which usually lead to high cost in large-scale experiments, although some recent works have addressed this dilemma at lower costs (14–16). By building one barcoded library for massive cells to analyze large amount of cells in parallel, droplet-based methods like Drop-seq and InDrop (17,18) are more efficient in sequencing, and the pioneer 10X Chromium Single Cell Gene Expression Solution sequencing platform (19) can even obtain the expression profile of up to 10 000 single cells at one time. However, compared with plate-based methods, they require specialist equipment, and because of the limited reading depth, fewer genes can be detected per cell (20–22). Due to the automated separation and mixed sequencing of cells, it is difficult to trace the specific source of each cell.

A core feature and also challenge of single cell RNA-seq expression profile (SCEP) data is its high dimensionality. In emerging researches and applications, large-scale single cell sequencing of complex tissues requires quantities of experiments and data analysis (23). Therefore, cost-effective data collection, storage and computation methods are of the essence, which leads to the idea of dimensionality reduction during data acquisition (24). In addition to high dimensionality, another important characteristic of scRNA-seq data is sparsity. In general, SCEP usually contains a large portion of zero values (25,26), that is, only relatively few genes are observed to be expressed in each cell. These zero counts observed may be due to low mRNA sequenced within individual cells, transient gene expression, or ‘dropout’ events by technical causes (27,28). The sparse feature of scRNA-seq data inspired the idea of applying data compression.

Given these features, some studies have developed methods to increase the number of samples by reducing sequencing depth in individual cells, called shallow RNA-seq (24,29). In other cases, limited number of genes are designed to be detected and counted, to guide and speculate the expression profile of those unmeasured genes together with earlier profiling data (30,31). These approaches reduce the dimension of SCEPs from the perspective of genes to achieve cheaper and easier data collection (32). However, since it is easy to obtain the expression values of most genes by using plated-based sequencing technologies, reducing the number of samples seems to be a more pivotal way. Is this a viable approach? No research has yet given this answer.

Compressed sensing (CS) theory is a signal processing theory for sparse or compressible signals (33). For these signals, less observation data can be obtained with low sampling, and then the original signal can be recovered with high probability through a non-adaptive reconstruction algorithm (34). Analog to sparse signals, single cell expression data also own sparse structures, indicating that it may be worthwhile to apply compressed sensing theory to SCEPs. If we compress the expression profile during experiments and infer the entire original signals by CS algorithm, the library costs of plate-based methods might be reduced while incorporating their advantages in gene detection sensitivity and cell identity tracing.

In this paper, we explored the compressibility of single cell expression data and the approach of applying CS inference framework to single cell expression measurements. The basic roadmap is to subsample different cells into overlapped pools, construct libraries for pools (less than the number of cells) to get composite sequencing results and then apply certain CS algorithms to recover expression level for each cell by the observed results. Since the conditions of pools with mixed cells are known, it could be possible to decompress the signal and recover the individual original expression values. Through computational simulations (35,36), we proved the feasibility of our approach and tested two kinds of regularization models (37,38). In practical experiments, combined with Smart-Seq2 library construction protocols, we inferred the SCEP of 54 human immune cells using the sequencing results of 28 overlapped pools. Compared with the results of conventional Smart-seq2 method, 48.15% (26/54) cost of library construction was saved, 86.46% genes were detected, and the mean Pearson correlation coefficient of 54 cells reached 0.875. In all, our results suggested a new research direction for the field of utilizing the compressibility of SCEP data.

## MATERIALS AND METHODS

### Computational simulation

#### Key resources

The key resources used in this work are listed in Table 1. A dataset of 64 human pancreatic islet cells (GSE73727) is considered as dataset1 which has high sparsity. Sixty-four human immune cells picked up from dataset GSE98638 were named as dataset2 as a contrast which has low sparsity. The 53 760 cells’ expression data of 7 mice were collected from https://doi.org/10.6084/m9.figshare.5829687.v7, and these cells were sorted by FACS and sequenced with Smart-Seq2 from 20 organs.

**Table 1.** Key resources table

DEPOSITED DATA	SOURCE	IDENTIFIER
Gene expression data	Single-cell transcriptomes reveal characteristic features of human pancreatic islet cell types (29)	GSE73727
Gene expression data	Landscape of infiltrating T cells in liver cancer revealed by single-cell sequencing (9)	GSE98638
Gene expression data	Single-cell transcriptomics of 20 mouse organs creates a Tabula Muris (14)	doi.org/10.6084/m9.figshare.5829687.v7

The inference procedure is designed to reconstruct a single cell expression profile by a known measurement matrix and the compressive measurements. As single cells are subsampled into overlapped pools, the sequencing results of each pool are considered as compressive measurements here.

It should be clarified that the gene expression matrix during reconstruction is the transposition of traditional gene expression matrix (each row of the expression matrix represents a gene and each column represents a cell), which doesn’t affect the result of sequencing.

#### Sparsity description

Dataset1 was sequenced based on plate-based Smart-Seq2 protocol, containing seven cell types: alpha cells, beta cells, delta cells, PP cells, duct cells, acinar cells and undefined cells. The morphology and anatomical regions of these types of cells are different, leading to large differences in their expression level. The correlation between cells is low with a median of 0.147. The non-zero value in dataset 1 only accounts for 24.49%, indicating that dataset1 is a relatively sparse matrix.

Dataset2 was selected from a large single cell dataset of 5063 cells and contains a total of 14 952 genes, including six cell types: PTC, TTC, PTH, TTH, PTR and TTR. Though these cells have different tissue sources and cell subtypes, they are all T lymphocytes with little morphological and functional differences. The correlation between cells in dataset2 is high, with a median value 0.738. Cells from same tissue or same cell type show higher similarity, such as PTR and TTR (both CD4+CD25highT cells). The proportion of non-zero values in dataset2 is 32.30%, which is significantly higher than that in dataset1, indicating that dataset2 has a low degree of sparsity. The visualized information can be seen in Supplementary Figure S1 and S5.

#### Measurement matrix generation

Measurement matrices $M$ are randomly generated Bernoulli matrices with dimensions of $pool{\rm{\ }}number \times cell{\rm{\ }}number$, where the probabilities of 0 or 1 to appear in a matrix are both 0.5 (the probability of occurrence of 1 is set as an adjustable parameter $p$, here, $p\ = \ 0.5$). The Bernoulli matrix is a commonly used measurement matrix in compressed sensing theory. When the measurement number of the random Bernoulli matrix satisfies $M \ge cKlog( {N/K} )$ (*c* is an extremely small constant), it will meet the RIP condition with great probability. In parallel computing scheme, for each sub ${M_i}$, we used the same method to generate measurement matrix as above.

When a number in a measurement matrix is 0, it means that the cell corresponding to it will not be subsampled, so the deviations in our method are mainly related to the cells added in the pool, which could be sampling error, sample degradation or sequencing error. Based on it, we build our turbulence model in computational simulation. When a turbulence is set as $t{\rm{\ }}( {t \in ( {0,1} )} )$, we replace value 1 with a random number sampled from a uniform distribution $u \in ( {1 - t,1 + t} )$.

#### Datasets example simulation processing

The gene expression matrix for 64 single cells of different sparsity (dataset1 and dataset2) were downloaded as $X$. The compressive measurements $Y$ were then built up by multiplying generated $M$ with $X$ ($Y\ = {\rm{\ }}M \times X$). Randomly generated $M{\rm{\ }}$was varied on the number of pools from 15 to 45 (interval = 5, repeat = 50). Turbulence was set as 0, 0.2, 0.4, 0.6. For dataset1 of high sparsity, we solved $\overset{\frown}{X}$ by ${\ell _1}$ regularization (l1-magic toolbox), which based on Basis Pursuit model. For dataset2 of low sparsity, we solved $\overset{\frown}{X}$ by ${\ell _2}$ regularization based on Ridge Regression by the following equation.

$$\begin{equation*}x\ = {\left( {\lambda I + {M^T}M} \right)^{ - 1}}\ {M^T}y\end{equation*}$$

$x,{\rm{\ }}y$ stands for each column of $X,{\rm{\ }}Y$. $\lambda$ is a trade-off between sparsity and accuracy. $\lambda$ was set to 0.01 in this article based on grid search results.

We used Pearson Correlation Coefficient to compare reconstructed $\overset{\frown}{X}$ with the original${\rm{\ }}X$. Though $X$ is solved column by column, we care more about reconstructed performance of each cell. So, we calculated the Pearson Correlation Coefficient by comparing inferential gene expression of each cell to its original data (${\rho _c}$), and we considered mean of Pearson Correlation Coefficient of different cells as the detection consistency of our sequencing method to Smart-Seq2 method ($\rho$) as follows:

$$\begin{equation*}{\rm{\ }}{\rho _c} = \frac{{\mathop \sum \nolimits_{i = 1}^n \left( {{x_{c,i}} - \bar{x}} \right)\left( {{{\hat{x}}_{c,i}} - \overline {\hat{x}} } \right)}}{{\sqrt {\mathop \sum \nolimits_{i = 1}^n {{\left( {{x_{c,i}} - \bar{x}} \right)}^2}\mathop \sum \nolimits_{j = 1}^n {{\left( {{{\bar{x}}_{c,j}} - \overline {\hat{x}} } \right)}^2}} }}\ \end{equation*}$$

$$\begin{equation*}\rho \ = \frac{{\mathop \sum \nolimits_{c = 1}^m {\rho _c}}}{m}\ \end{equation*}$$

#### Parallel comprehensive model on large dataset

We collected data of 5063 single cells from NCBI (GSE98638) and then excluded unqualified single cells followed the original paper for downstream analysis. To implement a parallel model, we first constructed ${M_i}$. To implement parallel scheme, we partitioned original expression profile by rows into sub groups of cells’ expression profile ${X_i}$, whose row number equals the column number of ${M_i}$. The row number of ${M_i}$ is the number of cells in each block, and column number is the number of pools in each block. Among them, the number of rows in each ${M_i}$ is 500 (563 in ${M_n}$, $n\ = \ 10$), and the number of columns is 158 (178 in ${M_{10}}$). Each block was then computed using same scheme as the two datasets above. Finally, we merged all recovered${\rm{\ }}\widehat {{X_i}}{\rm{\ }}$by rows to form the SCEP. For classification visualization, we chose 4034 cells from original dataset, which contains all NTC, PTC, TTC, NTH, PTH, TTH, NTR, PTR and TTR cells in the dataset.

The 53 760 cells’ expression data of 7 mice were collected from https://doi.org/10.6084/m9.figshare.5829687.v7, and these cells were sorted by FACS and sequenced with Smart-Seq2 from 20 organs (14). Same as the processing procedure of the article, we removed low-quality data, and 45 432 cells passed a QC cutoff of at least 500 genes and 50 000 counts. Genes that were not expressed in all cells were removed. We constructed ${M_i}$ with 500 rows, and segmented the expression profile $X$ by one for every 500 rows. The ${M_i}$ here is $500 \times 250$, and ${M_n}$ is $423 \times 200$ ($n\ = \ 91$). For the sake of simplifying the experimental operations, set $p\ = \ 0.3$. After each block was calculated, all of the blocks were merged. Due to the large differences within each organ type, cells of each type were clustered before inference to obtain a better cell sequence for final visualization.

## SINGLE CELL SEQUENCING EXPERIMENT

### Preparation and culture of cells

In this study, all plastic and glass consumables were properly sterilized, dried and UV-treated before use. A dedicated set of pipettors were cleaned with ethanol (Sigma Aldrich) every time before experiments. Especially in the experiments involving RNA, all work surfaces, pipettors and equipment were cleaned by RNaseZapTM (invitrogen) and rinse off with RNase-free water in advance, and all the disposable tubes are pretreated with 0.1% DEPC. All reagents were vortexed and spun briefly before use. Nuclease-free water (Ambion) was used in all experiments. Room temperature was kept at 25°C using an air conditioner during experiments.

Cell line GM12878 was purchased from Coriell Institute, and cell line HEL, U-937, HMy2.CIR, CEM/C1, HL-60 and Ramos were purchased from American Type Culture Collection (ATCC). Cells were cultured as the instruction on the corresponding official website (Coriell Institute and ATCC).

### Single cell isolation and library construction

The cultured cells were washed and resuspended in PBS (Mg^2+^ and Ca^2+^ free). Single cells were isolated by gently mouth pipetting into RNase-free tubes, then treated by SMART-Seq HT Kit (Takara), which is based on Smart-seq2 technology. Single cells were first lysed and then performed one-step first-strand cDNA synthesis and ds cDNA amplification in a thermal cycler. It is worth mentioning that the temperature cycles used in this verifying experiment are 23, which is higher than the recommended cycling number in manual (17–18 cycles). Because cDNA samples would be separated into two parts, one for subsampling by measurement matrix and one for conventional Smart-seq2 protocols as contrast, we increased cycles to ensure sufficient yield of cDNA. But when applying our single cell RNA compressed sequencing in real cases, since there is no need for extra cDNA sequencing by traditional methods as contrast, cycling number can be set as recommended in manual. The amplified cDNA was then purified using the Agencourt AMPure XP Kit and checked the quality by Agilent 2100 Bioanalyzer and Qubit fluorometer. The cDNA of 54 single cells was passed the validation. Part of purified cDNA was then prepared for sequencing library according to manual as traditional Smart-Seq2 method. The other cDNAs were subsampled into pools for the compressed sequencing.

### cDNA subsampling and sequencing

A measurement matrix $M$ with dimensions of $40( {pool{\rm{\ }}number} ) \times 54( {cell{\rm{\ }}number} )$ was generated in the same way we did in the computational simulation. If ${M_{ij}} = \ 1$, 4 ng cDNA from cell $j$ will be subsampled into pool $i$, otherwise if ${M_{ij}} = {\rm{\ }}0$, cDNA from cell $j$ will not be added to pool $i$. The mass of cDNA defined as a portion was calculated by the content of cDNA left after Smart-Seq2 library construction to make sure that cDNA of all single cells was sufficient for subsampling. After subsampling, 40 pools were prepared into sequencing libraries. The libraries of 54 single cells and 40 subsampled pools were then sequenced on HiSeq XTen using 2 × 150 paired-end reads.

### Read alignments and gene-expression estimation

Qualified sequencing reads from 54 cells and 40 pools were aligned to human (hg19) reference genome using STAR with default settings (39). Gene expression was calculated as TPM values for each transcript using RSEM (40). Gene expression matrix of 54 cells by Smart-Seq2 method could be directly acquired by RSEM results. To make sure gene expression values in 40 pools are comparable to them of 54 cells, we chose TPM value to normalize gene length and sequencing depth as comprehensive as possible. For sequencing data of pools, since several cells equally share the depth of a pool (the mass of each subsampled single cell cDNA is the same), the real depth of each single cells in this pool is the depth divided by the number of cells. To recover their depth to the same level of Smart-Seq2 data, an additional normalization step should be conducted as follows.

$$\begin{equation*}{\rm{\ }}{Y_{TPM}} = {\rm{\ }}diag\left( {\frac{1}{{rowsum\left( M \right)}}} \right)M{X_{TPM}}\end{equation*}$$

$$\begin{equation*}{\rm{\ }}{M_{normalized}} = \ diag\left( {\frac{1}{{rowsum\left( M \right)}}} \right)M\end{equation*}$$

It is noteworthy that we don’t need this normalization step in computational simulation because we obtain compressive measurements $Y$ there by matrix multiplication. Then ${\hat{X}_{TPM}}$ was solved by CS method from ${Y_{TPM}}$ and ${M_{normalized}}$. Because the samples we used were all human immune cells and most of them have certain diseases, we assumed that their gene expression are active and the sparsity of gene expression matrix is low. The non-zero value of the matrix accounts for 29.97%, which supports this conclusion. Here, we first performed CS inference using all of the 40 pools, and then we randomly selected 28 pools from 40 pools to further test the performance of the algorithm. We compared ${\hat{X}_{TPM}}$ with ${X_{TPM}}$ from Smart-Seq2 method in terms of gene detection sensitivity and consistency. Considering rounding errors resulted from floating point operations and biological significance of TPM values, we considered genes whose TPM values were below 0.001 as unexpressed or undetected genes, and compared the number of genes detected by different methods as gene detection sensitivity. The detection consistency was calculated as the same equation in computational simulation part by Pearson Correlation Coefficient.

## RESULTS

### Framework of Compressed sensing strategy

#### Compressibility of single cell expression data: theoretical basis

The application premise of compressed sensing theory is that the original signal is sparse. In practice, as long as the signal approximately satisfies the sparsity, that is, most of the values tend to zero, the signal can be considered as compressible and can be subsampled (41). As mentioned above, many zero values constitute SCEP into zero-flated data. Hence, the sparse feature of SCEP makes it suitable for the theoretical framework of compressed sensing.

In addition, the existence of relevancies among data is a major prerequisite for its compressibility. Regulatory modules in gene expression data have been proved and studied in many articles, which is due to the existence of co-expressed genes (42). They are co-regulated by certain transcription factors, expressed or silenced (43). To some extent, these modules bring a certain degree of data redundancy and further provide opportunities for the compression of single cell expression data. Some studies have utilized these modules to infer module activities and further ‘decompress’ or ‘reconstruction’ the expression level of individual genes (32,44,45).

Here, we deem the compressibility of single cell expression data also closely related to the heterogeneity level between samples in the dataset. If the characteristic genes of each type of cells differ considerably, there will be more protruding features for signal recognition. When compressing, it can better target at the less important ‘redundancy’, and more accurately grasp the significant information that can distinguish the data when restoring. For instance, the two datasets we use below vary in sparsity degree with different heterogeneity levels. Supplementary descriptions of this part can be found in the Materials and Methods section.

#### From grouping idea to solving linear equation: model design

**Figure 1.**
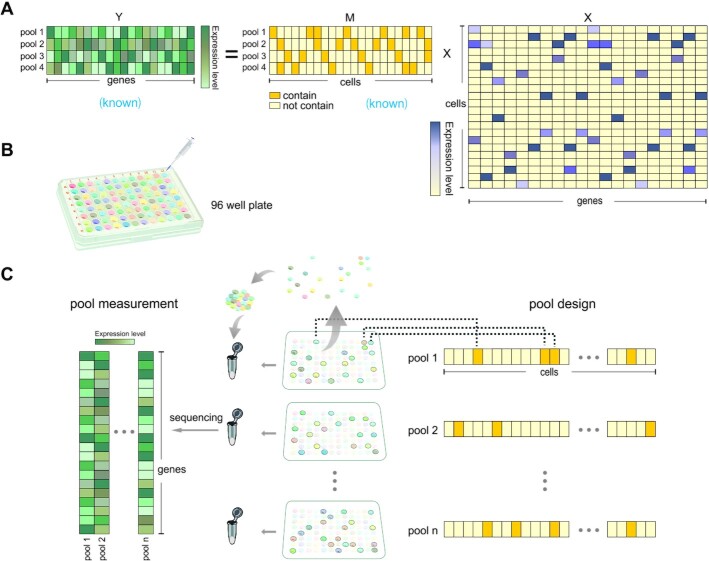
Objection overview illustration. (**A**) Illustration of pool strategy for single cell expression profile inference. The $M$ matrix is pool-cell matrix, which is overlapped. In this example, 22 cells are designated into 4 pools. ${M_{11}}$ is painted means pool1 contains cell1. $Y$ is pool-gene matrix, which is the expression level for each pool after sequencing. Different shades represent different levels. $X$ is cell-gene matrix, that we want to infer from $Y$ and $M$. It is usually sparse among most genes. (**B**) Illustration of pooling strategy, same amount of amplification products of each cell is extracted into pools. (**C**) In the middle, different masks on the 96-well plate represent different cell group in each pool. Each plate is an $8 \times 12$ matrix, on the right is a long one-dimensional vector with length of 96. It contains value zero (unpainted) and one (painted) indicating how to choose cells for each pool. On the left, a grouping-based sequencing result for each pool.

To find a systematic approach to achieve compressed sampling and reduce library usage, we learned the idea of group testing method, dividing all cells into overlapped groups. The ‘overlapped’ here means that a single cell appears in different pools, and one pool contains a group of cells. This overlapping fashion utilizes the same cell as an information bridge and can reduce the tests by cross information. Equation (1) is a representation of the theoretical model for our method (Figure 1A):

(1) $$\begin{equation*}{Y_{p \times g}} = {M_{p \times c}}{\rm{\ }} \times {\hat{X}_{c \times g}}\end{equation*}$$

Among them, $M \in {\mathbb{R}^{p \times c}}$ stands for the measurement matrix, a known matrix generated by computer in computational simulation. In single cell practice, since pooling (or grouping) is the central idea of our method, we designed the matrix $M$ artificially to divide different cells into different pools. In the basic scenario, $M\ = \{ {{m_{ij}}} \}$ is a binary $p \times c$ matrix, where $p$ indicates the number of pools which equals the number of library we will use, and $c$ represents the number of cells. Accordingly, ${m_{ij}} = {\rm{\ }}1$ if and only if ${i^{th}}$ pool contains ${j^{th}}$ cell (Figure 1A). Pools are designed according to the $M$ matrix first, and then sequenced using NGS technology. $Y \in {\mathbb{R}^{p \times g}}$ represents the observation data, which is the sequencing results of sub-sampling samples by pools. $\hat{X} \in {\mathbb{R}^{c \times g}}$ represents the inferred expression matrix, the desired SCEP, where $g$ is the number of genes detected. Suppose the original SCEP is $X \in {\mathbb{R}^{c \times g}}$, then ${Y_{:j}} = M \times {\hat{X}_{:j}}$ can be easily interpreted as: we get ${Y_{:j}}$ (a compressed format of gene $j$’s expression profile) by using a combination of detectors $M$ to overserve the SCEP ${\hat{X}_{:j}}$ (original format of gene $j$’s expression profile). To sum up, by means of combining the observed $Y$ and the measurement matrix $M$ to solve equation (1), the initial SCEP $X$ can be reconstructed as $\hat{X}$.

Experimentally, since every single cell will be sequenced many times, the aliquots of its cDNA amplification products can be used to represent the exact cell. Each pool in $M$ contains several cells, and each cell appears in different pools (Figure 1B and C). Suppose we want to sequence 96 unique cells using the pooling strategy, we would first add the amplification products of each cell into different wells of a 96 well plate (Figure 1B), and then adopt a different combination of different cells for each pool according to the pool design (Figure 1C). For each pool, the required cells are extracted into a test tube; next, one library is constructed for one tube and then sequence a whole tube at a time.

#### Model solving: other descriptions

After the observed $Y$ is obtained by random sub-sampling, the restore of the original data is an indispensable step in compressed sensing theory. Our goal is to solve equation (1) effectively in high accuracy with a properly designed $M$. In order to realize the signal reconstruction, not only the original SCEP $X$ should be compressible, but also the measurement matrix $M$ needs to meet certain conditions. Restricted Isometry Property (RIP) should be satisfied, which is equivalent with the proviso that the measurement matrix is not interrelated to the sparse representation base. To achieve this, our constructed $M$ needs to be as random as possible. The requirements on $M$ can be relaxed if we just want a silhouette of the SCEP.

Mathematically, the ill-conditioned linear equation (1) ($p$ is usually much less than $c$, which meets our needs to use less library) has infinite answers. However, compressed sensing can help to solve this equation when $X$ is sparse. In order to restore the original $X$ without overfitting, certain regularization requirements should be meet on the boundary conditions of equation (1). This is an optimization problem of the minimum ${\ell _0}$ norm. However, it is an NP-C problem, so it is difficult to be solved directly. Usually, it is converted to the minimum ${\ell _p}$ norm to solve. In this paper, considering the different sparsity characteristics of sample matrix, we applied two different regularization models, Basis Pursuit model (${\ell _1}$ norm) and Ridge Regression model (${\ell _2}$ norm) for data inference. ${\ell _1}$ regularization is inclined to get sparse solutions, while ${\ell _2}$ norm can make the solutions smoother, close to but not zero. The adaptability and effectiveness of these two models are compared and evaluated.

### *In silico* simulation of compressed sensing strategy

#### Inference of data with high sparsity

We first applied compressed sensing method on dataset1 (GSE73727) to evaluate the recovery ability. This dataset contains 7 types of cells, making up a total of 64 cells. After statistical analysis, we decided dataset1 a relatively sparse matrix (Supplementary Figure S1).

We inferred the primary SCEP and applied three measurements to visualize and evaluate the performance:

1. Mean Pearson correlation for 64 cells between the original SCEP and the inferred one. Two regularization models were used to complete the reconstruction, Basis Pursuit model and Ridge Regression model, to compare their restore accuracy.
2. A heat map of correlation for 64 cells, original and inferred, in a certain circumstance.
3. Correlations between the inferred genes and original genes, ranked by their sparsity level and expression value.

**Figure 2.**
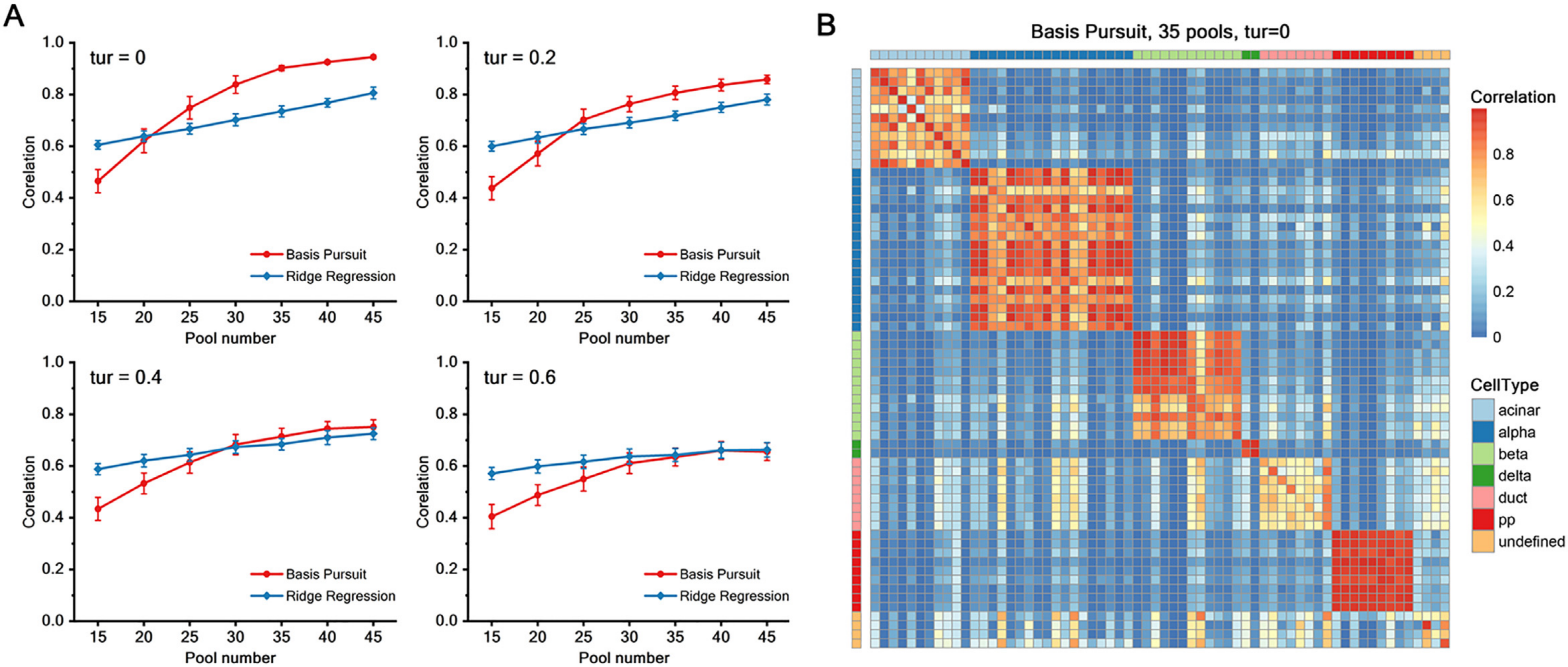
Simulation results of dataset1. (**A**) Mean Pearson correlation for cells in dataset1 between original and inferred SCEP with the Basis Pursuit model and the Ridge Regression model and with different pool numbers under different error perturbations. Repeat = 50, the error bar represents the standard deviation of the data. (**B**) A heat map of correlation for 64 islet cells between original data and its inference by Basis Pursuit model. 35 pools were used, no turbulence.

As expected, the overall correlation calculated between the reconstruction result and the original dataset rises with the increment of the pool number due to the increase in sampling times (Figure 2A). Meanwhile, as the perturbation increases, the correlation coefficient gradually decreases, and the dispersion of the result under random repetition expands. For these two models, when the pool number is large, the effect of reconstruction using Basis Pursuit model is normally better than that of using Ridge Regression model. The reduction effect of pool number under 20 is too low for practical applications. At the same time, it can be noticed that when the perturbation increases, the advantage of using Basis Pursuit model gradually falls because Ridge Regression model is more robust to perturbation. Under 0.6 turbulence, we can see that Ridge Regression performs better in every pool condition.

It’s worth noting that when the pool is set to 35, the mean Pearson correlation can still reach 0.908 (Basis Pursuit), which can save nearly half of the cost (29/64) of sample sequencing and library construction. And in general, using 35 pools can capture most information which is highly coherent with original data, visualized by heat map (Figure 2B). Every types of cells can be distinguished explicitly. A low-dimensional embedding representation for 64 cells (t-SNE) can also prove the results of the cell classification (Supplementary Figure S2A).

The restore performance of each gene can be seen in Supplementary Figure S4A. Consistent with the characteristics of ${\ell _1}$ regularization, we find that those genes expressed in fewer cells (this value defines sparsity level, that is, the fewer cells a gene is expressed in, the higher its sparsity level is) show great inference results, and their correlations with the original data almost reach 1. On the contrary, the reduction effect of dense genes expressed in most cells is mediocre. As the gene expression values (the accumulation of expression values in all cells) increase, their correlations gradually decrease from close to 1 to around 0.8, telling the same trend as ranked by sparsity level. Fortunately, we are more concerned with genes that are specifically expressed in certain cells or cell types than housekeeping genes.

#### Reconstruction of data with low sparsity

In order to probe which regulation model is more suitable for a less sparse dataset, we selected another 64-cell single-cell dataset2 (in contrast to dataset1) as the experimental data, including six cell types (GSE98638). The statistical data indicate that dataset2 has a low degree of sparsity (Supplementary Figure S1).

**Figure 3.**
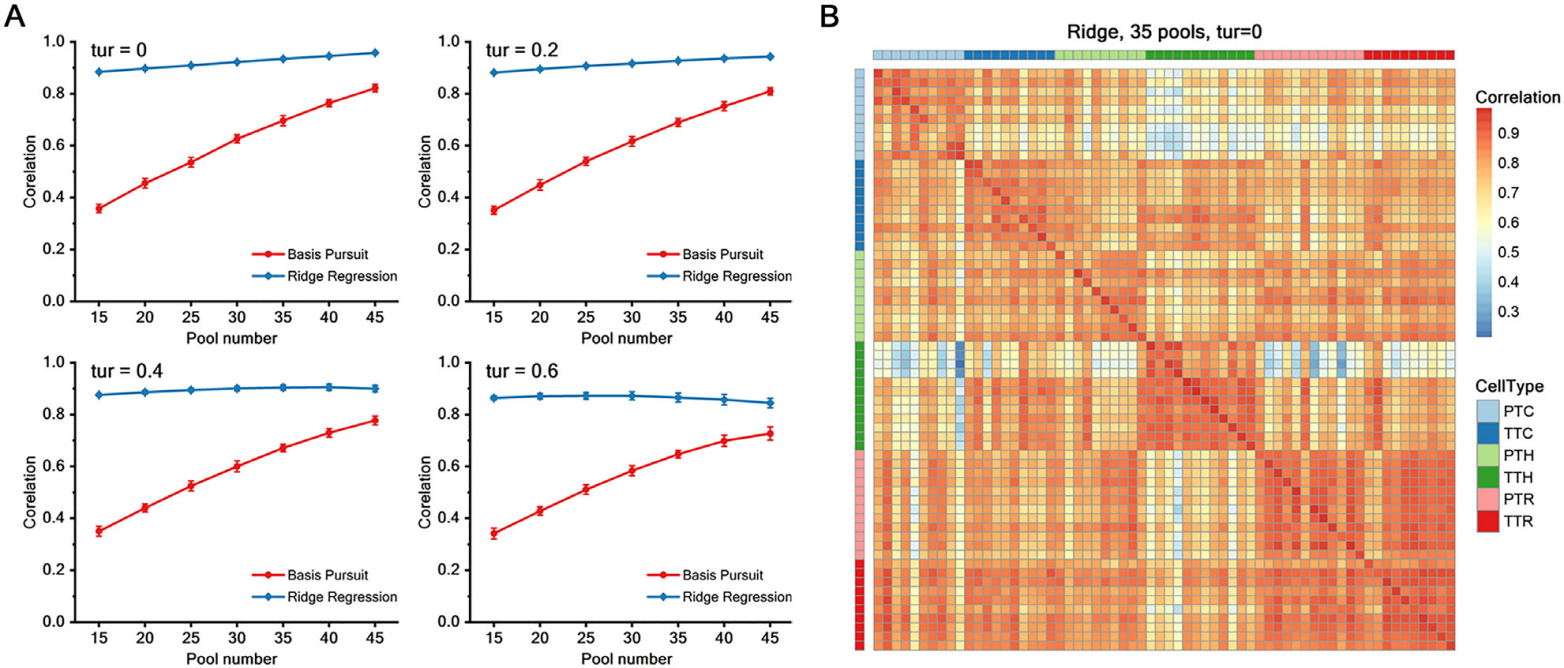
Simulation results of dataset2. (**A**) Mean Pearson correlation for cells in dataset2 between original and inferred SCEP with the Basis Pursuit model and the Ridge Regression model and with different pool numbers under different error perturbations. Repeat = 50, the error bar represents the standard deviation of the data. (**B**) A heat map of correlation for 64 lymphocytes between original data and its inference by Ridge Regression model. 35 pools were used, no turbulence.

As with dataset1, we performed SCEP recovery and the identical measurements to visualize and evaluate the regression performance. Differently, it can be seen from Figure 3A that the inference effect of dataset2 by Ridge Regression model is generally better than Basis Pursuit model in any condition, with the correlation coefficient reaching more than 0.85. The dispersion of the results is slightly affected by pool number (sampling times) and remains almost unchanged under 50 random simulations, indicating that the inference effect of Ridge Regression model is credibly stable. From the perspective of perturbation, Ridge Regression model also has a superior robustness. When the disturbance value is between 0 and 0.4, the difference of correlation coefficient results is very small, which may be related to the consideration of noise in data in the optimization part of Ridge Regression model.

Because of the little difference between the cells in dataset2, distinguishing them by their expression characteristics is a more difficult job. Seen from heat map result, the inferred SCEP using equally 35 pools can generally separate these cells into their own clusters, which is basically consistent with the original SCEP, especially PTC and TTH (Figure 3B). More sampling times are needed to present a fine t-SNE result (Supplementary Figure S2B).

Compared with violin plots of dataset1, as the sparsity level declines and the expression value rises, the correlations of genes in dataset2 show the similar downward trend (Supplementary Figure S4B). The difference is that, although the sparse genes’ correlation of dataset2 is not as high as that of dataset1, its overall situation is more stable. Furthermore, dense genes in dataset2 perform better, with a median correlation only a bit lower than 0.8. The nature of ${\ell _2}$ regularization to choose more non-zero features may provide explanations for these results.

Overall, these results for two datasets showed that by using compressed sensing method to subsampled expression matrix, people can successfully infer SCEP with high accuracy when pool number meets and regularization model fits. For data with high sparsity, Basis Pursuit model has a better inference performance, while Ridge Regression model is more suitable for data with low sparsity, and it is also more robust to disturbances. Therefore, we achieved the goal to cut down the library cost nearly twice. Though only half of the cost was cut down and more experimental efforts were paid, the merit of our approach will be amplified when dealing with much more cells. However, challenges are still remained for recovering dataset with an enormous scale of single cells. Next, we will extend this method to tackle both problems.

#### Parallel comprehensive model on large dataset

We focused on a dataset with 5063 T cells isolated from different tissues of liver cancer patients (GSE98638) as a showcase. Our comprehensive parallel model was applied on these cells using the same reconstruct strategy as dataset1 and dataset2.

In calculation process, since Ridge Regression model can be solved based on analytical solutions and does not require iterative approximation, its calculation speed is significantly superior to that of Basis Pursuit model, gaining advantages in the calculation of large amounts of single-cell transcriptome data.

We manipulated the simulation from 1600 pools which is over three times less than 5063 libraries as before. To achieve the goal of reducing calculating time and increase the inference accuracy, we divided the original SCEP into small blocks (500 cells per block, 10 blocks in total) instead of building a large conversion matrix, and restored each block using the previous strategy in parallel, and finally merged all the restored results together. For better visualization, 4034 cells were selected from the 5063-cell dataset.

**Figure 4.**
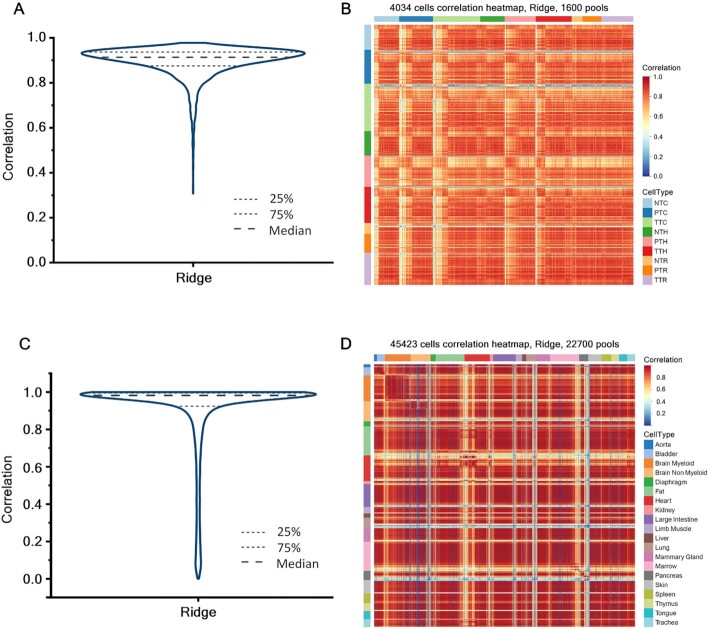
Visualization of the inference performance for large dataset. (**A**) The correlation distribution diagram of using the Ridge Regression model to infer original 4034 cells expression profile. (**B**) Pearson correlation heat map between 4034 cells and their inferences. (**C**) The correlation distribution diagram of using the Ridge Regression model to infer original 45 423 cells expression profile. (**D**) Pearson correlation heat map between 45 423 cells and their inferences.

As illustrated in Figure 4A, over 75% cells have correlation coefficients over 0.80 with their inferences, which accumulate around 3200 cells. In addition, our model successfully distinguished nine different cell types by using only sparse genes (Figure 4B). Each type of cells shows different expression features, which proves that most of the information of original data can already be obtained by using only 1600 pools in experiment.

Next, to explore the application value of this parallel model on a much larger dataset, we selected a larger dataset with a scale of 53 760 cells (14). This dataset contains approximately 10 times the amount of data in the previous dataset, and its characteristics are different from the expression profile of human cells, since its data come from 20 types of mouse organs. After removing the low-quality cells, we obtained a 45 423-cell SCEP. After obtaining the inferred SCEP (22 700 pools used), we also observed the heat map and violin plot made from the correlation values of all cells and compared them with Figure 4A and B.

According to Figure 4C, we can see that the inferred correlations of the vast majority of cells are close to 1, and more than 75% of the cells have correlations >0.95. Meanwhile, Figure 4D also shows the expression patterns of each type of cells, and the diagonal lines are clearly visible, which is similar to the results in Figure 4B. This result indicates that the original SCEP can be roughly restored with half of the cell number, which is of guiding significance for large-scale single cell sequencing experiments.

In practical experiments, we can use this parallel idea to group cells first. Combine the difficulty of operations with the overall number of cells to determine the number of cells in each group, such as 100 to 500 cells per group. For each group of cells, implement mixing and sequencing operations, and perform compressed sensing inference. Experiments of these groups can be carried out simultaneously and finally the obtained results will be combined. In this way, processing large-scale cells will become feasible.

### In vitro verification experiment of compressed sensing strategy

After computer simulation, we carried out practical experiments to test the feasibility of our method. For stress testing, we cultivated 54 human immune cells (7 cell types with small differences) as experimental samples and obtained corresponding cDNA samples. According to the compressed sensing measurement matrix generated by the computer, we subsampled and mixed the samples to obtain 40 cDNA mixing pools. Finally, 54 single cell samples and 40 sub-sampling mixed pools were sequenced. We performed bioinformatics processing on the scRNA-seq data and obtained 54 single-cell samples with high mapping rates, with an average of 93.40%. After TPM normalization of the gene expression matrix, we removed the genes expressed in none of the cells, and got 21 375 genes remained. The alignment rates of the 40-pool data were higher and their fluctuations were little, with an average of 97.44%. TPM normalization was also performed.

**Figure 5.**
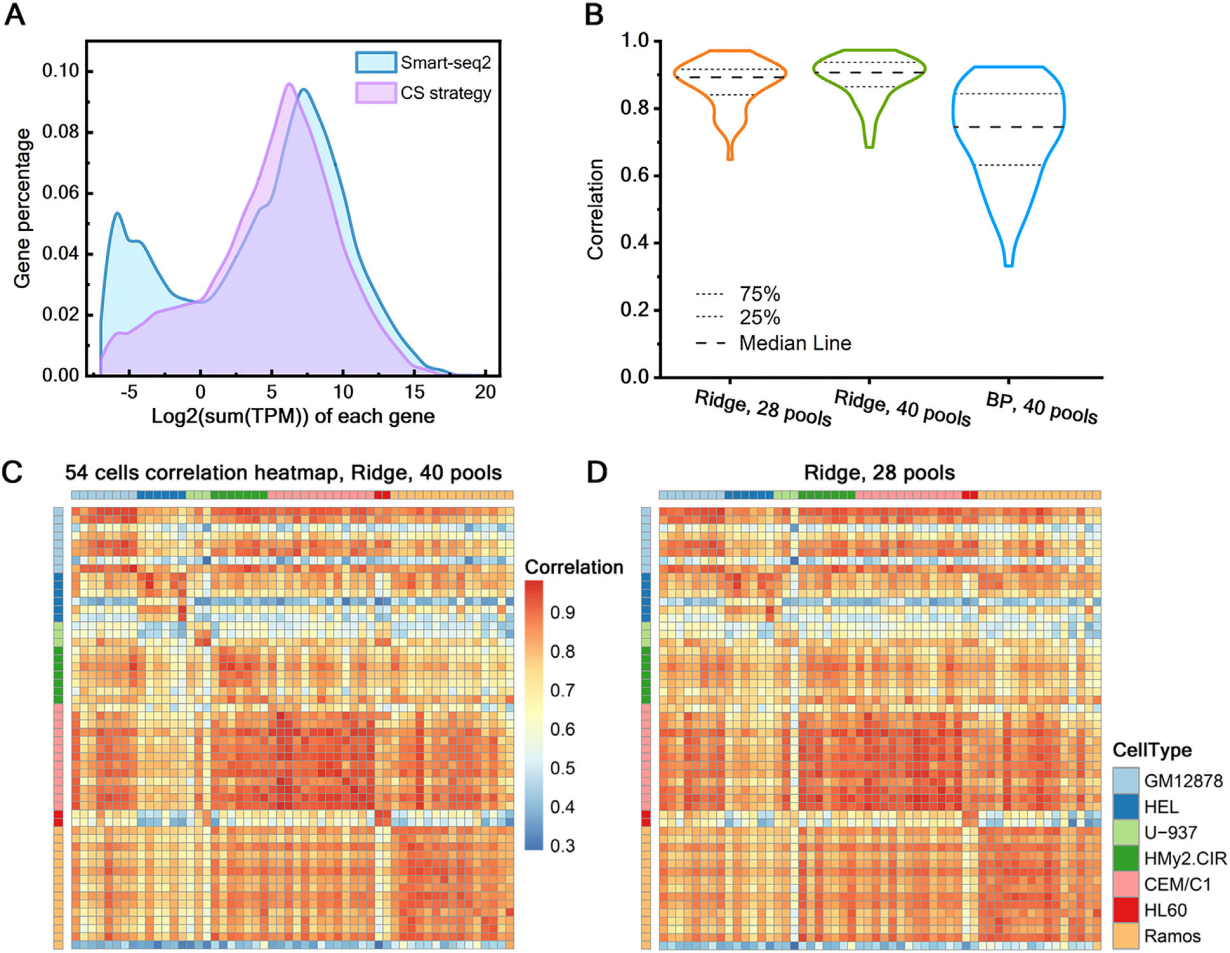
*In vitro* verification results with experimental data. (**A**) The gene distribution diagram of SCEP by original results and inferred results (the *x*-axis is ordered by the sum of TPM value for each gene, and the y-axis represents the percentage of the number of genes in the total 21 375 genes). (**B**) The correlation distribution diagram, a comparison of Ridge Regression with 28 and 40 pools, and Basis Pursuit model with 40 pools. (**C**and **D**) A heat map of correlation for 54 lymphocytes between original results and inferred results. Ridge Regression model, 40 and 28 pools used respectively.

After inspection, we found this dataset had low sparsity (Supplementary Figure S3A), and the distribution of genes with different sparsity levels also shows its closeness to the profile of dataset2 (Supplementary Figure S5). This result manifested that the subsampled data in this experiment were more suitable for Ridge Regression model to infer the original expression profile. After successful inference with 40 pools (Figure 5C), we extracted 28 pools randomly from 40 pools for inference to test the possibility of reducing more cost using this method. Genes with a sum of TPM value <0.001 were removed, with a total of 18 245 genes obtained. After compressed sensing reconstruction by Ridge Regression model, we obtained the inferred SCEP of 54 cells with a 54 × 18 245 matrix. Compared with the traditional Smart-seq2 result (dimension 54 × 21 375), there were 3130 genes less, accounting for 14.64%. In addition, we set the expression values of genes whose TPM value is <5 to 0 to ensure the accuracy, and finally got the median of the expressed gene number per cell as 6678. In summary, in this experiment, the genes detected in total reached 85.36% of that of the traditional Smart-seq2 method, and only 1.82% of the undetected genes were genes with TPM values >1 (Figure 5A).

The inferred SCEP obtained by Ridge Regression reconstruction was compared with SCEP by Smart-seq2, and the cell-to-cell correlations were visualized (Figure 5B). The average coefficient of 54 cells’ correlation is 0.875, with a median of 0.892. Besides, the standard deviation of the data is only 0.069. SCEP reconstruction was also carried out using the Basis Pursuit model, and it was found that its correlation with Smart-seq2 SCEP was significantly lower than that of the Ridge Regression model, even if 40 pools were used, and the former degree of dispersion is high (Figure 5B).

As with the verification measurements above, we examined the performance of reconstruction by drawing a heat map of the Pearson correlation between Smart-seq2 and inferred SCEP. As Figure 5D displayed, the numerical results for each cell of inferred results using Ridge Regression model is generally similar to the traditional scRNA-seq results, with high correlation coefficients on the diagonal. Furthermore, some cells can already distinguish their cell types, such as CEM/C1 and HL60 cells. Judging from the performance of gene reconstruction, the results are acceptable (Supplementary Figure S4C). We can observe that most of the results are consistent with the simulation results of dataset2, but the overall median values of the correlation coefficients are lower. Emphasis should be placed on the sparse or low-expressed genes in experimental data. Their correlations between primary genes are not high, which is most likely resulting from perturbations in the experimental process, such as sample contamination and mRNA degradation. Due to their specificities, measurement errors on them will affect the inference results to a greater extent. Correspondingly, genes ranked 20–40% perform best, reaching a median value close to 0.7. When the number of pools reaches 20, the overall correlation shows more errors, and the inferred cell identities are unauthentic to believe (Supplementary Figure S3B). Based on these results, we believe 28 a reliable pool number, indicating nearly half of the library cost save.

Given the above, using compressed sensing strategy for scRNA-seq has high gene detection sensitivity and SCEP inference accuracy. Not only in computational simulations, but also in real experiment operations, this method proved its feasibility. Because of the stability fluctuation and various constraints in practical operations, Ridge Regression model has more application value in most cases and especially real single cell experiments. In addition, normalization of data is also necessary for this strategy in practical. We must be clear that the pool expression values we sequenced display the sum of all single cell expression in the pool. As speculated, in the experiment of our study, Ridge Regression method is more suitable for the inference of SCEP. Moreover, through this strategy, we can save a lot of library cost and data processing time, source the identities of cells and even predict the disturbance level of the scRNA-seq data, which has a wide range of application prospects.

## DISCUSSION

In this study, we explored the compressibility of single-cell expression data. In addition to mathematical modeling and computer simulation, we proved through experiments that the SCEP directly obtained from data collection can be compressed from the perspective of cells. To achieve this process, we compressed sampled the expression profile by overlapped pooling strategy and inferred original signals using compressed sensing theory. Here, we listed some important thoughts on this framework.

We believe that the proposal of this approach is instructive in certain respects. In the first place, by using Smart-seq2 protocol, our results retain the identity information of each sample and guarantee high gene detection sensitivity, which massive droplet-based methods cannot do; we can tell the expression information of each cell, which indicates that this method is useful for areas such as spatial transcriptomics. Moreover, given that highly varied genes contain the most crucial information for classification, the recovery performance of our method for such genes has been able to capture the structural gist of the original profile. Finally, at the most basic level, we reduced the number of libraries by nearly half, which may save a mass of money when sample size is large. From the financial perspective, our method may be an economic alternative to these plate-based single cell sequencing methods.

For this method, the comparison of the two regularization models in compressed sensing algorithm played an indispensable role. Through two different types of datasets, we found these two models have their own advantages and are suitable for different scenarios. In principle, for datasets with high sparsity, choosing Basis Pursuit model (${\ell _1}$ regularization) will get better solutions, yet Ridge Regression model (${\ell _2}$ regularization) performs better when the sparsity is low. At the same time, Ridge Regression model exhibits better robustness under turbulences, so it can be more suitable for most situations, just like our experimental results demonstrated. In fact, In the scenario of scRNA-seq, many inevitable disturbances will be introduced during sample loading manually and cDNA degrading naturally. Therefore, the expression matrix of single cells is difficult to be really sparse, leading to inaccurate numerical solutions. As seen from Supplementary Figure S5, dataset with high sparseness contains most genes expressed in few cells and almost no genes expressed in all cells, showing a perfect descending distribution curve, whereas our experiment data shows a rise in dense genes proportion. We recommend that a small-scale sampled sequencing can be performed to roughly predict the overall sparsity level before implementing CS strategy on new samples.

Though the cell correlation between our results and original data keeps at a high level, the CS strategy still comes with limitations in real experiments. (i) Experimental measurements will inevitably include noise. Whether in the process of mixing cells or sequencing, the emergence of errors will exert a certain impact on the accuracy of the inference model. There is always a trade-off between numeric precision and experimental efforts. This is also the case when we want to increase the number of pools for better performance. The automatic sampling systems might relieve this dilemma between performance and experimental efforts in the future, and also providing more possibilities for large-scale experiments. (ii) Since the current design is not exquisite enough, the frame itself contains systematic errors. First, SCEP shows a negative binomial distribution whereas our methods model the data as the prior distributions of regularization models. Second, due to the restriction of compressed sensing algorithm, it is difficult to accurately restore the expression level of dense genes without any reference or assumption. Besides, information loss during decompressing results in data distortion to a certain extent, while the process of regularization tends to make the solutions of linear equations more ‘averaged’, which may be the main reason for the inaccuracy.

For future improvements, we envisage changes in the following directions. Similar with co-regulated genes, cells from same individuals, tissues or positions, or with similar phenotypes display similar expression patterns. If the dependencies between cells could be used to form cell modules that serve as pre-information for low rank compressed sensing recovery, it might lead to better performance of dense genes recovery, or require less overlapped pools for sequencing. Meanwhile, optimization of the algorithm is necessary before our method can be widely used, such as employing superior regularization models.

To sum up, SCEP analysis is an emerging and exciting area to explore with its intrinsic features like sparsity. These features, in turn, bring opportunities and challenges, requiring novel statistical and computational methods. Based on the sparse feature of scRNA-seq data, our work offered a comprehensive study on inferring the original signals from the compressed sampled expression profile. This framework may provide a technical guide to other datasets of biological information with similar structures, including data from proteomics, lipidomics and metabolomics, etc. We believe that more works in this area are enthusiastically to be seen for the coming years.

## DATA AVAILABILITY

Scripts to implement Compressed Sensing inference are available in the GitHub repository (https://github.com/Easteryang/CS_inference).

The sequencing data of 54 single cells and 40 mixed pools in the experiment have been deposited with Sequence Read Archive (SRA) database under accession number PRJNA684992.

## Supplementary Material

gkab581_Supplemental_File
