# Supplementary material for: Inferring single cell expression profiles from overlapped pooling sequencing data with compressed sensing strategy
Source: Nucleic Acids Res. 2021 Jul 9;49(14):7995–8006. doi: 10.1093/nar/gkab581 (PMC8373083; doi:10.1093/nar/gkab581)
Supplement: gkab581_Supplemental_File [file gkab581_supplemental_file.pdf]

Supplementary Materials

Supplementary Figures

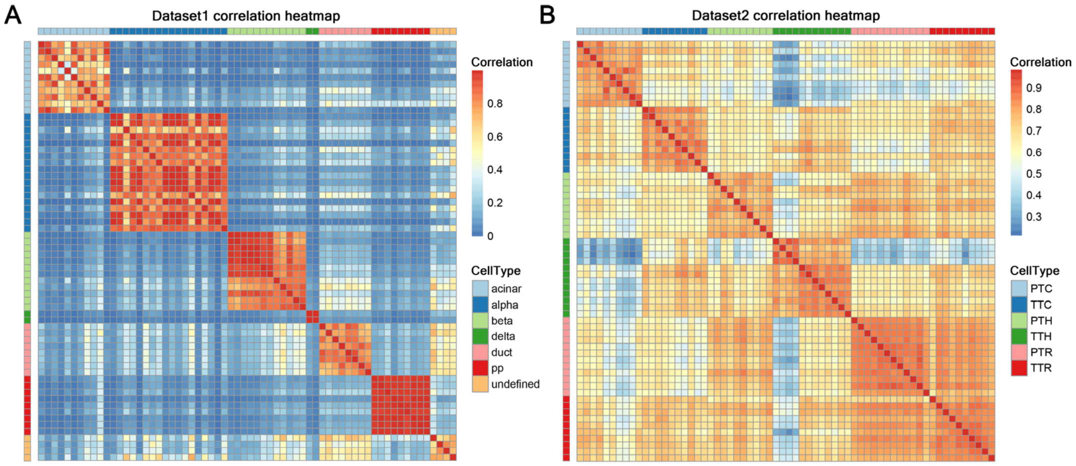

Figure S1. Pearson correlation heat map between cells in dataset1 and dataset2.

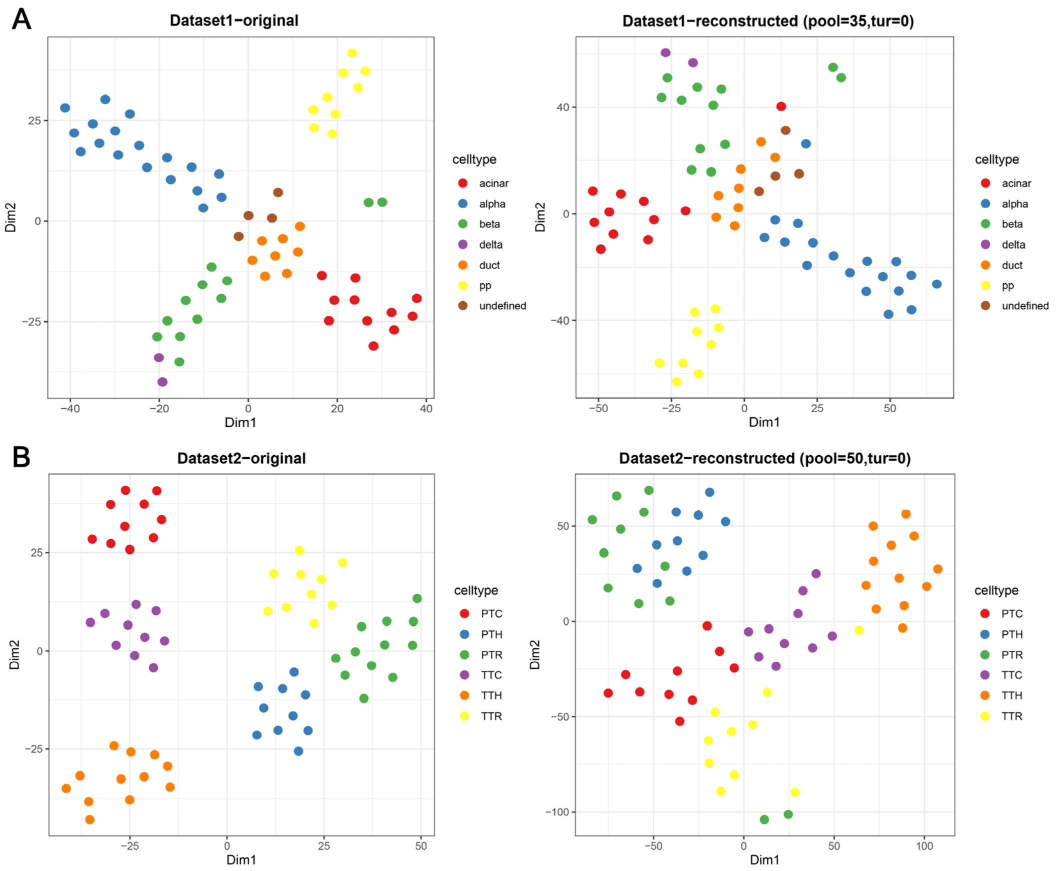

Figure S2. (A) t-SNE visualization for both original data and its inference by Basis Pursuit model for 64 cells in dataset1. 35 pools were used, no disturbance. (B) t-SNE visualization for both original data and its inference by Ridge Regression model for 64 cells in dataset2. 50 pools were used, no disturbance.

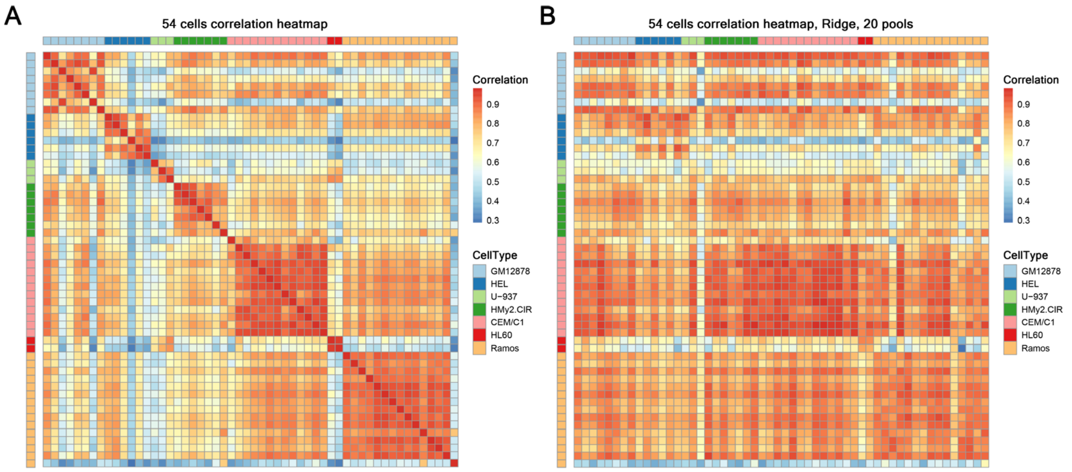

Figure S3. (A) Pearson correlation heat map between 54 lymphocytes in single cell experiment dataset. (B) Heat map of correlation for 54 lymphocytes between Smart-seq2 and comRNA-seq results. Ridge Regression model, 20 pools used.

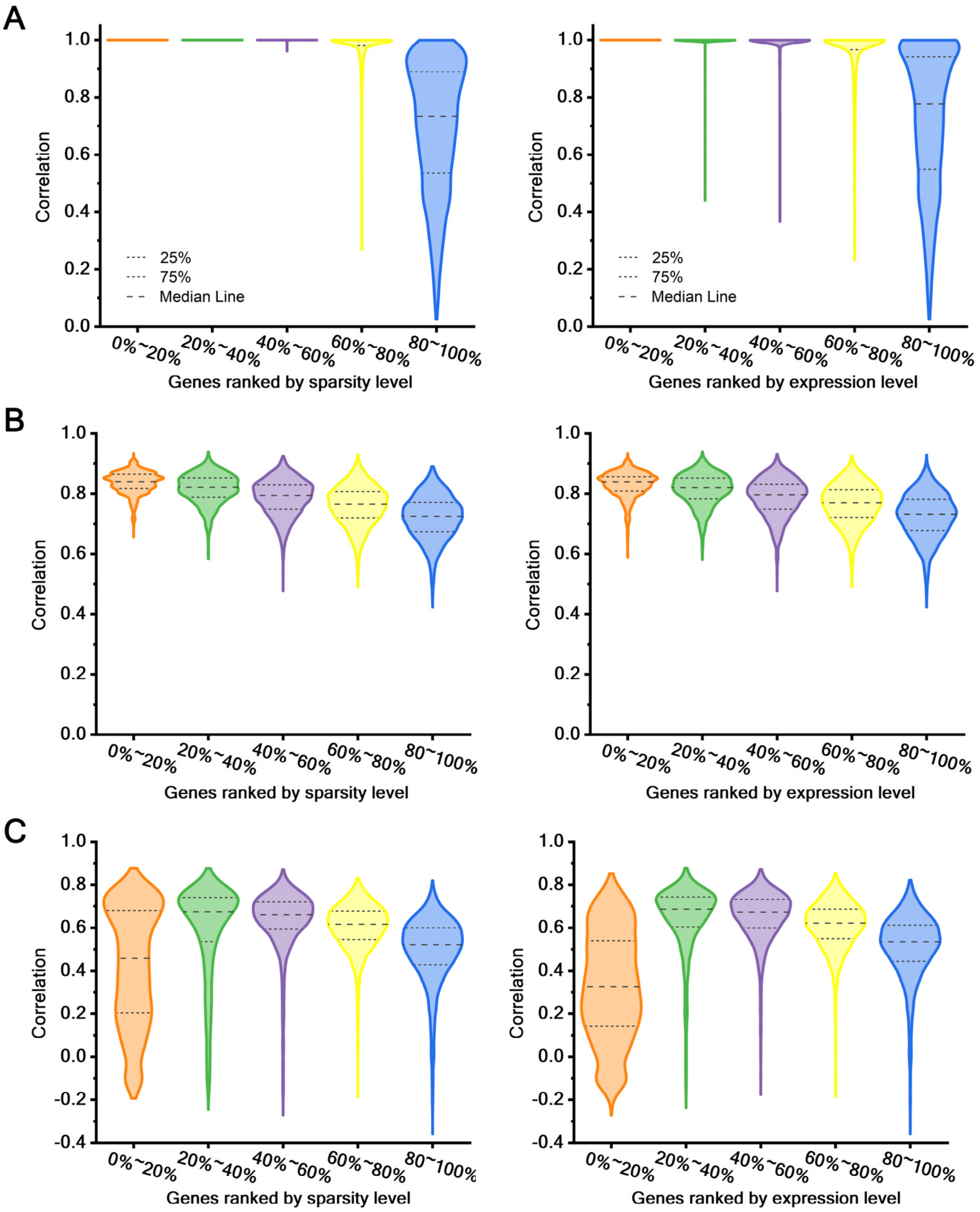

Figure S4. Violin plots of Pearson correlations between the inferred genes and original genes, ranked by their sparsity level (left) and expression level (right). (A) stands for results of dataset1, (B) for dataset2, and (C) for experiment dataset. For each gene, the fewer number of cells it expresses in, the higher the gene sparsity level is; the expression level is calculated by its sum of expression values in all cells.

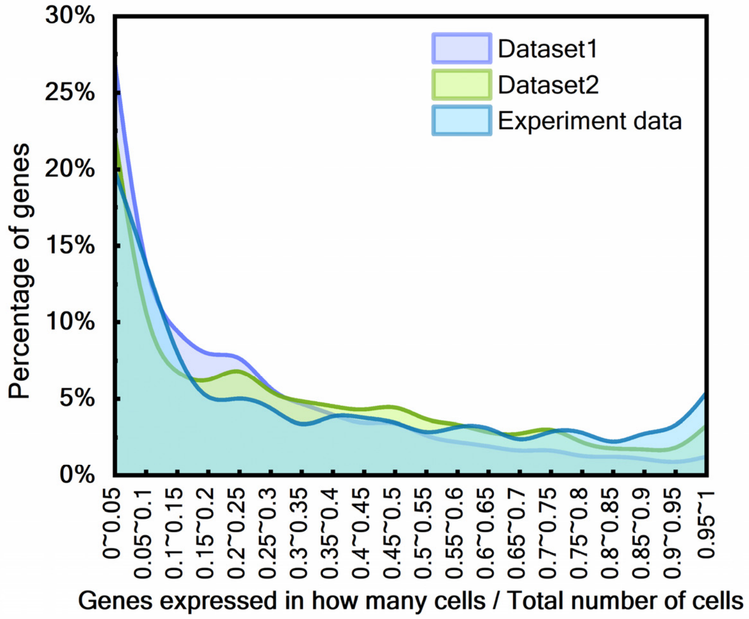

Figure S5. The distribution of genes with different sparsity levels in dataset1, dataset2 and single cell experiment dataset.
